# Supplementary material for: Designing and Evaluating a Health System Resilient to Extreme Weather Events in Rural Madagascar
Source: Ann Glob Health. 2025 Jul 22;91(1):40. doi: 10.5334/aogh.4759 (PMC12292047; doi:10.5334/aogh.4759)
Supplement: Supplementary Table S1. — Results of GLMM models evaluating the impact of Batsirai on long-term trends in indicators. UHC-tracer availability and consultation rates at CHWs were not complete enough in the recent-HSSi zone to compare between zones. Coefficients represent either rate ratios or exponentiated average monthly change (trends). 95% CIs are shown in parentheses and those CI which do not overlap 1 are bolded. [file agh-91-1-4759-s1.pdf]

Table S1. Results of GLMM models evaluating impact of Batsirai on long-term trends in indicators. UHC-tracer availability and consultation rates at CHWs were not complete enough in the recent-HSSi zone to compare between zones. Coefficients represent either rate ratios or exponentiated average monthly change (trends). 95% CI are shown in parentheses and those CI which do not overlap 1 are bolded.

	Trend (slope over time)	historic-HSSi	Trend x historic-HSSi	Change in trend post-intervention (recent-HSSi only)	Change in intercept post-cyclone	Change in trend post-cyclone	Post-cyclone intercept x historic-HSSi
Malaria case rate	**1.04 (1.03 - 1.05)**	**3.33 (1.79 - 6.17)**	**0.98 (0.96 - 0.99)**	0.94 (0.85 - 1.04)	0.92 (0.6 - 1.39)	1.07 (0.96 - 1.19)	0.73 (0.4 - 1.33)
Malaria RDT positivity rate	1.01 (1.01 - 1.02)	0.64 (0.37 - 1.1)	0.99 (0.98 - 1)	**0.86 (0.8 - 0.93)**	0.89 (0.61 - 1.3)	1.19 (1.1 - 1.29)	0.68 (0.4 - 1.14)
Diarrhea case rate	0.99 (0.99 - 1)	1.83 (1.03 - 3.23)	1 (0.99 - 1.02)	**1.13 (1.05 - 1.21)**	1.01 (0.71 - 1.43)	**0.89 (0.83 - 0.95)**	1.03 (0.65 - 1.66)
Proportion of consultations with diarrhea	**0.99 (0.98 - 0.99)**	0.94 (0.67 - 1.33)	1 (1 - 1.01)	1.07 (0.99 - 1.15)	1.16 (0.75 - 1.81)	0.93 (0.86 - 1)	1.43 (0.83 - 2.47)
Consultation rate at CSBs	**1.01 (1.01 - 1.02)**	**2.98 (2.01 - 4.41)**	1 (0.99 - 1.01)	1.17 (1.1 - 1.24)	0.93 (0.7 - 1.24)	**0.86 (0.8 - 0.92)**	0.77 (0.52 - 1.15)
Referral rate	**1.02 (1.01 - 1.04)**	**13.67 (3.72 - 50.32)**	0.99 (0.98 - 1)	1 (0.94 - 1.07)	1.17 (0.84 - 1.62)	0.97 (0.91 - 1.04)	0.77 (0.53 - 1.11)
DTP3 vaccination coverage	0.99 (0.99 - 1)	1.06 (0.77 - 1.45)	1 (1 - 1.01)	0.98 (0.92 - 1.04)	1.21 (0.87 - 1.7)	1.04 (0.98 - 1.11)	0.85 (0.55 - 1.3)
UHC-tracer availability	**0.98 (0.97 - 0.98)**	NA	NA	NA	**0.8 (0.66 - 0.98)**	**1.02 (1.01 - 1.04)**	NA

Consultation rate at CHWs	**1.02 (1.01 - 1.03)**	NA	NA	NA	1.05 (0.82 - 1.36)	**0.96 (0.93 - 0.98)**	NA
Severe malnutrition intake rate	**1.07 (1.02 - 1.12)**	NA	NA	NA	1.97 (1 - 3.87)	1.06 (0.97 - 1.16)	NA
