# Supplementary material for: Designing and Evaluating a Health System Resilient to Extreme Weather Events in Rural Madagascar
Source: Ann Glob Health. 2025 Jul 22;91(1):40. doi: 10.5334/aogh.4759 (PMC12292047; doi:10.5334/aogh.4759)
Supplement: Supplementary Table S2. — List of medicines included in the Madagascar Ministry of Health Universal Health Coverage Tracer (UHC-Tracer) medicines for primary health centers (2019–2024). [file agh-91-1-4759-s2.pdf]

Table S2. List of medicines included in Madagascar Ministry of Health Universal Health Coverage Tracer (UHC-Tracer) medicines for primary health centers (2019-2024).

01 Albendazole, Tablet (400 mg)
02 Amoxicilline, Syrup Powder (250 mg)
03 Amoxicilline, Gel capsule (500 mg)
04 Amoxicilline and Clavulanic acid, Powder for injectable solution (1 g/ 200mg)
05 Beclomethasone, Oral Inhalation
06 Benzylpenicillin, Powder for injectable solution (1 000 000 UI)
07 Cotrimoxazole, Tablet (120 mg)
08 Cotrimoxazole, Tablet (480 mg)
09 Domperidone, Table (10 mg)
10 Iron + Folic Acid, Tablet (240 mg)
11 Gentamicine, Injectable solution (80 mg)
12 Hydrocholorothiazide, Tablet (10 mg)
13 Ibuprofen, Tablet (200 mg)
14 Metoclopramide, Tablet (10 mg)
15 Metronidazole, Tablet (250 mg)
16 Nicardipine, Tablet (20 mg)
17 Paracetamol, Tablet (500 mg)
18 Phenobarbital, Tablet (50 mg)
19 Zinc + Oral Rehydration Salts
